# Pulmonary Cystic Echinococcosis: A Ruptured and Infected Cyst Presenting As Pyopneumothorax

**DOI:** 10.7759/cureus.62003

**Published:** 2024-06-09

**Authors:** Tithi S, Pankaj Wagh

**Affiliations:** 1 Respiratory Medicine, Jawaharlal Nehru Medical College, Wardha, IND

**Keywords:** intercostal drain, contrast-enhanced computed tomography, albendazole, pulmonary cystic echinococcosis, pyopneumothorax

## Abstract

In this report, a case of 62-year-old female is described who came to the hospital with chief complaints of breathlessness and productive cough with salty whitish expectoration, which she had for two months, along with fever and right-sided chest pain, for three days. The case was identified as a ruptured pulmonary hydatid cyst with pyopneumothorax using contrast-enhanced computed tomography and chest X-ray. This was further supported by the *Echinococcus* antibody IgG test. Right thoracostomy, the placement of an intercoastal drain, and four days of continuous aspiration of 750 ml of serous fluid were used for managing the case. Following this, oral albendazole was used as a conservative measure.

## Introduction

Pulmonary cystic echinococcosis, a zoonotic illness, is typically caused by the larval phase of *Echinococcus granulosus*. Dogs and wolves are the primary carriers, with sheep, cattle, and deer serving as intermediate hosts. Humans become accidental intermediate hosts of the infection when they ingest food tainted by eggs released in dog feces [1-3].

Life cycle in humans

Dogs are infected with the tapeworm *Echinococcus granulosus*, which becomes cystic formations when the tapeworm eggs are swallowed by an intermediate host (usually sheep) as they graze. Following their consumption by predatory canines, the cysts complete their life cycle, and many tapeworms thrive inside the definitive host's gut. By ingesting afflicted foods that were contaminated with the eggs from the definitive host's feces, humans accidentally become intermediate hosts. Cysts are harbored within an accidental host by this, mainly in the liver through the portal vein and the system of lymphatics via the intestinal wall; Nonetheless, echinococcal cysts have the potential to develop in almost all bodily regions, with the exception of hair, teeth, and fingernails [4].

Depending on the size and location of the cyst, pulmonary cystic echinococcosis symptoms and signs might arise due to the mass effect produced, that is, compression of surrounding tissues, hemoptysis, coughing, shortness of breath, retention pneumonia, atelectasis, and congestion of the superior vena cava. Additional complications from pulmonary hydatid cysts include rupture of the cyst, secondary infection, suppuration, and pneumothorax [5].

## Case presentation

A 62-year-old thin-built, female patient presented with complaints of breathlessness and productive cough with salty whitish expectoration for eight weeks along with low-grade intermittent fever and right-sided chest pain radiating to the right shoulder for three days. She had no history of chronic respiratory disease, tuberculosis, hypertension, or diabetes mellitus. Chest auscultation revealed the presence of right-sided basal creps.

**Figure 1:**
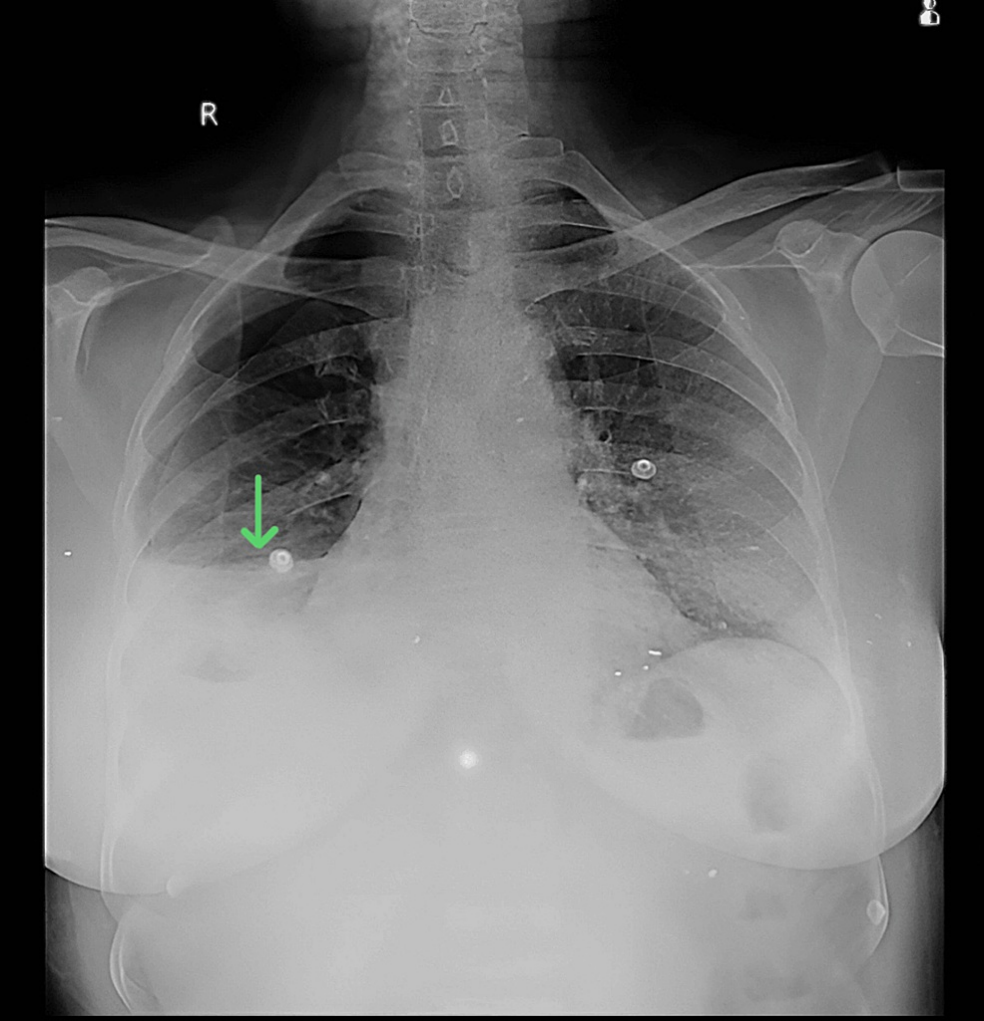
A supine anteroposterior chest X-ray showing right-side hydropneumothorax

 Anteroposterior chest X-ray in a supine position demonstrated homogeneity on the right side, along with air-fluid levels suggestive of hydropneumothorax (Figure 1).

**Figure 2:**
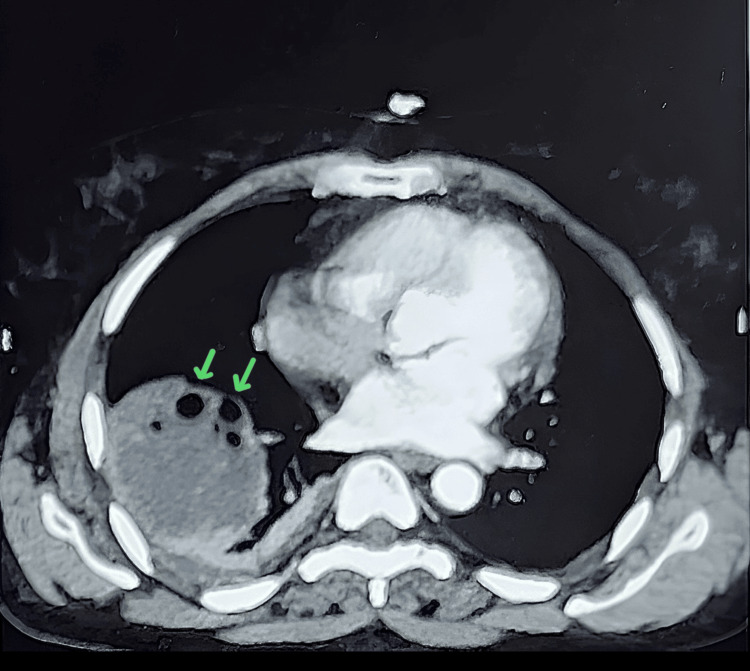
Contrast-enhanced computed tomography scan showing a well-defined lesion containing multiple air foci in the right lower lobe of the lung

A contrast-enhanced computed tomography scan revealed a well-defined heterogeneously hypodense lesion of 61 x 58 x 47 mm, involving the right lower lobe of the lung. The lesion also contains multiple air foci within and communicates with the anterior segmental bronchus of the right lower lobe (Figure 2). Radiological findings were suggestive of a ruptured pulmonary hydatid cyst along with mild to moderate right pleural effusion.

**Figure 3:**
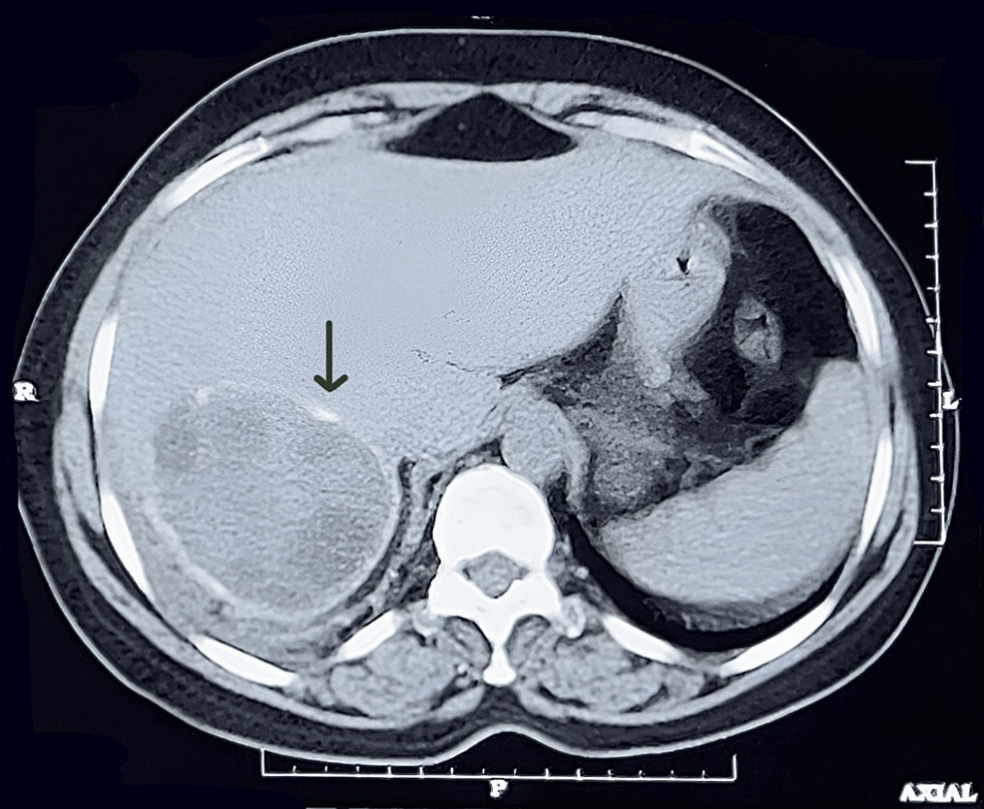
Contrast-enhanced computed tomography scan showing a well-defined lesion involving segments VI and VII of the liver containing a few daughter cysts

Moreover, a well-defined hypo-dense non-enhancing lesion of approximately 61 x 65 x 57 mm involving segments VI and VII of the liver. The lesion shows multiple peripheral coarse calcifications. It contains few daughter cysts, and the lesion is an exophytic component (Figure 3).

An *Echinococcus* granulosus IgG antibody test was subsequently performed on the patient, and the results turned out to be positive, which supported our diagnosis of pulmonary cystic echinococcosis.

**Figure 4:**
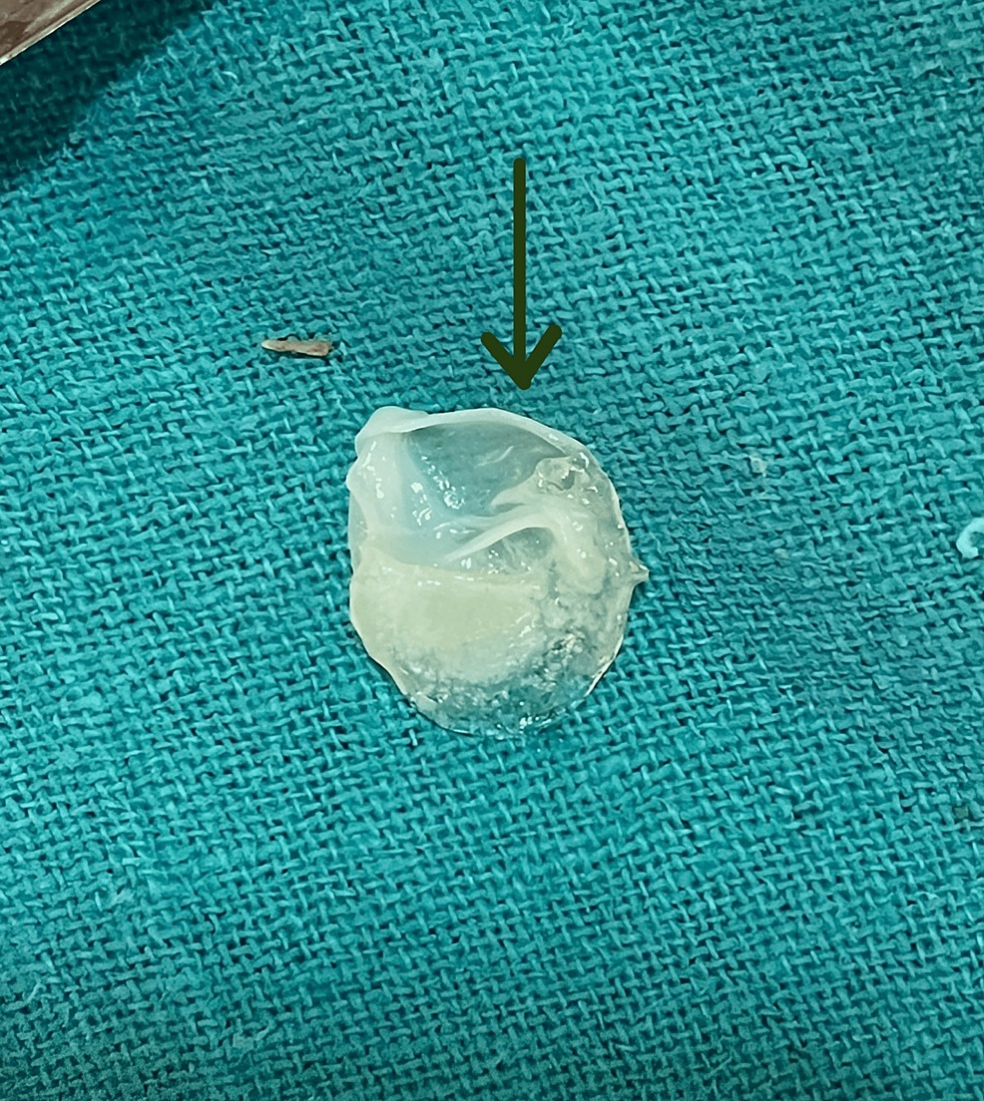
Ruptured pulmonary hydatid cyst aspirated through the intercostal drain

An intercostal chest drain was inserted after a right thoracotomy, and pyogenic fluid was aspirated continuously for four consecutive days. The aspirated fluid has a yellowish tint and pyogenic consistency, suggestive of pyopneumothorax. Along with the fluid, a ruptured pulmonary hydatid cyst, measuring 5 mm in diameter, also aspirated through the intercostal drain (Figure 4).

The patient was treated with ceftriaxone + sulbactum 1.5 gm twice a day. Albendazole 400 mg is given once every day for four weeks, followed by a two-month follow-up period. The pulmonary hydatid cyst completely disappeared, and all symptoms resolved.

## Discussion

Hydatid cysts are prevalent in rural areas due to close contact with dogs and sheep, making them endemic in certain regions of the world. The liver is where hydatid cysts are most frequently seen (75%), followed by the lung (15%) and other locations within the human body. In the posterior lower lobe compared to the anterior location, hydatid pulmonary cysts are more prevalent. Unruptured or uncomplicated cases are mostly diagnosed by accident and have limited or no symptoms. Cystic rupture can induce symptoms such as chest pain, cough, hemoptysis, fever, and shortness of breath, whether caused by trauma or spontaneously. The imaging process is necessary for the diagnosis, which is bolstered by serological and histopathological analysis. For assessing the location and size of a cyst, contrast-enhanced computed tomography is the most effective method. A crucial determinant of whether a cyst is parasitic or nonparasitic is its wall density [6,7].

Several names exist for the radiological signs of a ruptured hydatid cyst, including the whirl sign, iceberg sign, serpent sign, cumbo or double-arch sign, and sign of the rising sun. An air-fluid level is observed when air breaches the parasite membranes, causing the endocyst to collapse. The "Camelot sign" or "water lily sign," which resembles water lily leaves, is created if the parasite membranes hover over the fluid surface. The host-produced pericyst, also referred to as the "empty cyst sign," is all that is left after the parasitic contents have all been aspirated or discarded [8]. In addition to leucocytosis and an elevated erythrocyte sedimentation rate, both of which are frequently seen in instances with ruptured hydatid cysts, fewer than one-quarter of the individuals affected show an increase in eosinophils. However, these conventional evaluations lack specificity and may yield raised findings in a variety of distinct conditions [9,10]. Primary lung hydatid cysts may spontaneously rupture or become infected, or they may present with no symptoms at all. Surgical cyst drainage, germinative membrane peeling, and, in cases where surgical treatment is not feasible, albendazole therapy are available choices. The location of a hydatid cyst in the lung is important for determining the best course of action. Cyst draining and capitonnage are surgical procedures for centrally located cysts because the cyst may cause bronchopulmonary tree involvement. It is preferable to use lobectomy, combined albendazole treatment, wedge resection of the lung, and endocystectomy for asymptomatic cysts surrounded by parenchyma. Albendazole (10 mg/kg body weight) can be used as a medical treatment for four weeks in certain circumstances. Using them for at least three months can help minimize the number of scolices [11].

## Conclusions

To summarize, a ruptured pulmonary hydatid cyst may trigger anaphylactic reactions or can be asymptomatic. Albendazole treatment has worked effectively for the patient in this instance, but whenever feasible, parenchyma-sparing surgical methods ought to be employed. Certain common treatments for hepatic cystic echinococcosis are not recommended for pulmonary cysts. The aforementioned case report highlights the possibility of a zoonotic lung as a causative element for pleural effusion, hence indicating its inclusion as one of the potential causes.
